# Forgiveness and Moral Development

**DOI:** 10.1007/s11406-016-9727-6

**Published:** 2016-07-05

**Authors:** Paula Satne

**Affiliations:** Teaching Fellow in Philosophy, University of Durham (UK), 50, Old Elvet, DH1 3HN Durham, UK

**Keywords:** Forgiveness, Moral development, Imperfect and perfect duties, Self-reform, Humanity, Self-respect

## Abstract

Forgiveness is clearly an important aspect of our moral lives, yet surprisingly Kant, one of the most important authors in the history of Western ethics, seems to have very little to say about it. Some authors explain this omission by noting that forgiveness sits uncomfortably in Kant’s moral thought: forgiveness seems to have an ineluctably ‘elective’ aspect which makes it to a certain extent arbitrary; thus it stands in tension with Kant’s claim that agents are autonomous beings, capable of determining their own moral status through rational reflection and choice. Other authors recognise that forgiveness plays a role in Kant’s philosophy but fail to appreciate the nature of this duty and misrepresent the Kantian argument in support of it. This paper argues that there is space in Kant’s philosophy for a genuine theory of forgiveness and hopes to lay the grounds for a correct interpretation of this theory. I argue that from a Kantian perspective, forgiveness is not ‘elective’ but, at least in some cases, morally required. I claim that, for Kant, we have an imperfect duty of virtue to forgive repentant wrongdoers that have embarked on a project of self-reflection and self-reform. I develop a novel argument in support of this duty by drawing on Kant’s theory of rational agency, the thesis of radical evil, Kant’s theory of moral development, and the formula of humanity. However, it must be noted that this is a conditional duty and Kant’s position also entails that absence of repentance on the part of the wrongdoer should be taken as evidence of a lack of commitment to a project of self-reflection and self-reform. In such cases, Kant claims, we have a perfect duty to ourselves not to forgive unrepentant wrongdoers. I argue that this duty should be understood as one of the duties of *self-esteem*, which involves the duty to respect and recognise our own dignity as rational beings.

## Section I

Forgiveness has increasingly attracted the attention of moral philosophers and there is now an extensive literature on the topic. This is not surprising, given the human predisposition to wrongdoing and the importance of forgiveness for maintaining human relationships. Forgiveness, as a positive response to wrongdoing, should have a place in any convincing moral theory. Yet Kant seems to have very little to say about forgiveness. The *Groundwork* and the second *Critique* do not touch on the subject directly, but a relatively brief passage on the issue can be found in the Doctrine of Virtue in the *Metaphysics of Morals*, where Kant develops the more applied and causistical side of his moral theory.^1^ Perhaps for this reason, the topic of forgiveness has not attracted much commentary from Kant scholars and philosophers working in the Kantian tradition. On the one hand, Sussman (2005a) explains these omissions by arguing that there is a “deep ambivalence” (p. 88) in Kant’s treatment of forgiveness in the passage of the *Metaphysics of Morals*. Immediately after claiming that we have a duty to be forgiving, Kant warns us against its excess. An excessive readiness to forgive might manifest a lack of self-respect and a violation of a duty to oneself (Kant 1991, 460–1). Sussman claims that this ambivalence is unsurprising given that forgiveness sits uncomfortably in Kant’s moral thought: forgiveness seems to have an “ineluctably elective aspect” (p. 90) which makes it to a certain extent arbitrary and dependent on particular features of the forgiver’s psychology. However, Sussman alleges that this dependence is in tension with Kant’s claim that agents are autonomous beings, capable of determining their own moral status through rational reflection and choice. On the other hand, philosophers that work on forgiveness, but do not necessarily identify themselves as Kantian scholars, sometimes read the relevant passage of the *Metaphysics of Morals*, in which Kant claims that we have a duty to be forgiving, “partly because a man has enough guilt of his own to be greatly in need of pardon” (Kant 1991, 460/p. 253), as implying that we should forgive each other unconditionally because we are all to a certain extent evil in the sense that our guilt depends to a larger extent on moral luck (Murphy 1988, pp. 96–103; Garrard and McNaughton 2003, p. 55).

In this article I aim to show that there is space in Kant’s philosophy for a genuine theory of forgiveness and also lay the grounds for a correct interpretation of this theory. I argue that for Kant we have an imperfect and conditional duty of virtue to forgive wrongdoers, however, when the relevant conditions are not met, forgiveness is morally impermissible (Kant 1991, 460–1). I also offer a novel reconstruction of the Kantian argument for the derivation of this duty. Against Sussman I will argue that, at least from the Kantian perspective, forgiveness—far from being ‘elective’— is morally required and thus does not sit uncomfortably in Kant’s moral thought.^2^ Against Garrard and McNaughton and Murphy’s interpretations, I will show that the Kantian duty to forgive is not unconditional and that the argument that these authors ascribe to Kant is not only philosophically implausible but actually in tension with Kant’s central claims about freedom and agency. This paper offers a *reconstruction* of Kant’s position, one that provides a plausible interpretation of Kant’s texts but also develops some Kantian themes a bit further. I will proceed as follows. In section II, I introduce the relevant passage (Kant 1991, 460–61) in order to clarify Kant’s definition of forgiveness, noting some limitations and some advantages of his approach. In section III, I explain and reject two interpretations of the Kantian argument for the duty to be forgiving. In section IV, I start to develop a reconstruction of Kant’s argument for the derivation of the duty to be forgiving by briefly explaining some central features of Kant’s practical philosophy. These lend support to the view that the duty to be forgiving is not unconditional. I will draw on Kant’s theory of rational agency, the thesis of radical evil, and his theory of moral development. In section V, I complete the argument by deriving the duty to be forgiving from Kant’s formula of humanity and explain the central features of the Kantian duty to be forgiving as an imperfect duty of virtue. In section VI, I claim that for Kant we also have a perfect duty to ourselves not to forgive unrepentant wrongdoers (Kant 1991, 461). Section VII offers a brief conclusion and some directions for future research.

## Section II

Kant’s discussion of forgiveness can be found in the Doctrine of Virtue at the end of the section about the duties of love to other men. Kant divides the duties of love into the duties of beneficence, gratitude, and sympathy. Kant explicitly identifies ‘a duty to be *forgiving* (*placabilitas*)’ (Kant 1991, 461) as an imperfect duty of virtue that involves overcoming the vice of malice, which is the ‘direct opposite of sympathy’ (Kant 1991, 460). I first clarify and assess Kant’s definition of forgiveness. It is important to quote the relevant passage in full:

The sweetest form of malice is the *desire for revenge*. Besides, it might even seem that one has the greatest right, and even the obligation (as a desire for justice), to make it one’s end to harm others without any advantage to oneself.

Every deed that violates a man’s right deserves punishment, the function of which is to *avenge* a crime on the one who has committed it (not merely to make good the harm that was done). But punishment is not an act that the injured party can undertake on his private authority, but rather an act of a court distinct from him, which gives effect to the law of a *supreme authority* over all those subject to it; and when (as we must in ethics) we regard men as in a rightful condition *but in accordance only with laws of reason* (not civil laws), then no one is authorized to inflict punishment and to avenge the wrongs sustained by men except Him who is also the supreme moral lawgiver; and He alone (namely God) can say ‘Vengeance is mine; I will repay.’ It is, therefore, a duty of virtue not only to refrain from repaying another’s enmity with hatred out of mere revenge but also not even to call upon the judge of the world for vengeance, partly because a man has enough guilt of his own to be greatly in need of pardon and partly, and indeed specially, because no punishment, no matter from whom it comes, may be inflicted out of hatred. It is therefore a duty of men to be *forgiving (placabilitas)*. But this must not be confused with *meek toleration* of wrongs (*mitis iniuriarum patientia*), renunciation of rigorous means (*rigorosa*) for preventing the recurrence of wrongs by other men; for then a man would be throwing away his rights and letting others trample of them, and so would violate his duty to himself. (Kant 1991, 460–1/p. 253)

Kant thinks of forgiveness as a *personal* response to wrongdoing which consits in overcoming malice understood as a hateful desire for revenge. Kant adds ‘not even to call upon the judge of the world for vengeance,’ which presumably means not even to desire the wrongdoer to suffer disproportionally. The passage also establishes a clear separation between forgiviness and punishment. Althought Kant endorses a form of moral retributivism by claiming that violations of rights deserve punishment of the wrongdoer, he immediately adds that punishment cannot be inflicted by a private authority, but only by a court of a supreme authority. In the case of wrongs that are also legal offences, the supreme authority is the state, in the form of the courts. In contrast, violations of ethical laws, that is, moral wrongs considered *qua* moral, cannot be punished by anyone, including the courts (see also Kant 1991, 312)—except (we might hope) by the supreme authority of God (see also Kant 1998, 73). Forgiveness is not the forgoing of punishment, because punishment by a private individual or group is never allowed and punishment by the state is required, but only for wrongs that are also legal offenses (Kant 1991, 331). The issue of establishing (and implementing) how much suffering is proportionate to moral wrongdoing is a matter for God, not human beings. Thus, the passage defines forgiveness as an individual’s private response to wrongdoing, which involves overcoming emotions of hatred and vindictiveness and the forgoing of the desire for the wrongdoer to suffer disproportionally.

On the issue of the definition, Kant’s account has strengths, but also some weaknesses. One difficulty is that it could seem too narrow to limit forgiveness to the overcoming of hatred and vindictiveness. One difficulty is that the last section of the passage suggests that ‘meek toleration of wrongs’ would constitute a violation of a human being’s duty to herself (see also section VI); but if certain forms of forgiveness are not permissible, and thus, if one has a duty to oneself not to forgive in certain cases, then it becomes difficult to see how Kant could recommend that in some cases the right thing to do would be to hate another as a form of revenge. Moreover, the claim that by refusing to forgive in some situations we help to ‘prevent the recurrence of wrongs by other men by rigorous means’ suggests that there is more to forgiveness that the mere overcoming of vindictiveness. One could improve on Kant’s account^3^ by inviting a broader reading of these passages, allowing forgiveness to be understood more generally as the overcoming of various negative emotions, including vindictiveness, but also anger and resentment, which are usually felt by the victim in response to having been wronged. This would bring Kant’s account in line with the more standard (and widely accepted) definition of forgiveness usually attributed to Bishop Butler, which takes forgiveness as the overcoming of hostile emotions towards the wrongdoer, including hatred, anger, vindictiveness, and crucially resentment (Bishop Butler 1827). On this broader definition, forgiveness involves the overcoming of a variety of negative emotions of which resentment is perhaps the most interesting. Resentment is sometimes understood as a self-regarding form of anger caused by having been injured or harmed by a morally responsible agent (Hieronymi 2001).

One strength of Kant’s account is that his theory of rational agency puts him in a very good position to provide what Hieronymi has called an ‘articulate account of forgiveness’ (2001, p. 530). There are, of course, many competing philosophical accounts of emotions, but the problem is that, according to some conceptions, emotions are not under our immediate volitional control.^4^ But if emotions are beyond our control, then the possibility of forgiveness, understood as the overcoming of certain negative emotions felt towards the wrongdoer, becomes problematic. First, it seems that if emotions are beyond our control, then forgiveness is not an act that can be performed at will (Novitz 1998, p. 308) and the duty to forgive or cultivate a forgiving character might seem misguided. Second, too much emphasis on the involuntary character of the emotions has often forced philosophers to see forgiveness as a purely psychological matter requiring the ability to manipulate oneself out of an unpleasant state. However, in an important contribution to the topic, Hieronymi (2001) has argued that if forgiveness (and resentment) admit of justification, i.e. if there are good reasons to forgive (or resent) others, then “forgiveness will entail more than figuring out how to rid oneself of certain unfortunate affects” (p. 530). So, for example, taking a pill to get rid of one’s negative emotions would not count as a form of forgiveness. Instead Hieronymi urges that “genuine forgiveness must involve some revision of judgement or change in view … it must be an articulate account” (ibid.), and this in turn requires that we do not understand resentment and anger as things to be manipulated but “rather as attitudes sensitive to one’s judgements … [and] subject to rational *revision*” (pp. 534–5). She appeals to Scanlon’s notion of ‘judgement sensitivity’ (1998, pp. 20–24), and claims that we typically have attitudes like resentment and anger because we think we have a reason to have them.

Kant, of course, does not provide such a strong cognitivist account of the emotions, and to a certain extent he sees emotions as being partially outside our volitional control.^5^ But of course for Kant, actions are under our volitional control, so this suggests that emotions themselves are not direct triggers for action. Instead, according to what Allison (1990) has termed Kant’s Incorporation Thesis (IT), incentives (including all empirical motives and thus the emotions) influence the will by being incorporated into maxims (Kant 1998, 24). The IT entails that the negative emotions that forgiveness should overcome cannot be seen as forces to be dissipated through manipulation or as being themselves directly responsive to reasons. Instead the task for the agent is to decide whether or not to endorse these emotional responses by incorporating them into her maxims. As we will see in section V, the duty to be forgiving is an imperfect duty of virtue. Duties of virtue are primarily duties to have certain ends and to adopt, correspondingly, certain maxims. The duty to be forgiving is a duty to adopt a maxim of forgiveness and, since maxims are principles of justification, forgiveness on the Kantian account is paradigmatically responsive to reasons.

Some contemporary authors have noted that overcoming the negative emotions commonly associated with wrongdoing might be neither necessary nor sufficient for forgiveness (Scarre 2004, p. 25; Neblett 1974). Among the possible further conditions for forgiveness, authors have proposed reconciliation and full restoration of relationships, the forgoing of punishment, a more positive attitude of good will (or even love) towards wrongdoers (Garrard and McNaughton 2003, p. 44), and reintegrating the wrongdoer into the moral community. Given the variety of conditions that might be involved in forgiveness, Geoffrey Scarre has suggested that we should not attempt to provide a definition of the concept. Instead, forgiveness should be taken as a broad and varied family of practices (2004, p. 31). Scarre’s suggestion seems well-founded, but I believe that Kant’s account can admit a certain degree of flexibility. The passage currently under consideration (Kant 1991, 460–1) makes clear that for Kant, forgiveness and punishment are two separate issues, so a Kantian account of forgiveness would not demand the forgoing of punishment. But given that the duty to be forgiving is a duty to adopt a forgiving maxim,^6^ Kant’s account can accommodate the idea that in different situations forgiveness would involve a variety of forgiving practices, including the overcoming of negative emotions usually felt towards wrongdoers but also reconciliation and restoration of relationships, reintegration into the moral community, and other practices.^7^

The fact that forgiveness is an imperfect duty of virtue, which recommends the adoption of a forgiving maxim, has important implications for the Kantian account that I am developing. It is important to characterise this duty as duty to be forgiving, rather than a duty to forgive. The Kantian duty to be forgiving, thus, has some affinities with what Robert C. Roberts (1995) has called ‘forgivingness,’ which he characterises as the virtue of forgiveness, namely a disposition to abort one’s anger by seeing wrongdoers in benevolent terms provided by characteristic considerations of forgiving (1995, pp. 289–290). Similarly, for Kant, to adopt a forgiving maxim is to cultivate a forgiving character, that is, a willingness to forgive wrongdoers under circumstances that are deemed appropriate.^8^ Kantian ethics, thus, does not invoke a duty to forgive wrongdoers, but rather a duty to develop a forgiving character by adopting a forgiving maxim.

## Section III

I now want to consider and reject two recent interpretations of Kant’s views on forgiveness. In the passage, Kant only offers the following two considerations in support of the duty to be forgiving. We have a duty to be forgiving “partly because a man has enough guilt of his own to be greatly in need of pardon” and “partly … because no punishment, no matter from whom it comes, may be inflicted out of hatred.” In the second remark Kant reminds us that punishment inflicted out of hatred would be unjust, a matter of mere vengeance. This consideration, however, only provides support for a limited form of forgiveness, one that recommends that punishment be assessed objectively and dispassionately (Sussman 2005a, p. 89). The first remark is more substantive and has therefore received more attention in the literature,^9^ since some authors have read the passage as implying that because we are all guilty (that is, evil), then we should forgive each other (Murphy 1988, pp. 96–103; Garrard and McNaughton 2003, p. 55). Clearly, the passage admits such a reading. However, if this is Kant’s point, then it seems to me like a *non-sequitur*: we are all *so* bad (and note the implausibility of ascribing an equal degree of badness to everyone: we might all have moral flaws, but not the same ones and it is unlikely that we are all capable of committing the same sort of evil acts—I will come back to this point below) that we should *prima facie* be prepared to forgive each other as if wrongdoing is what should be expected from creatures like us. But if we are all bad, we might as well not forgive anyone. Perhaps the idea is that we should all forgive each other because we are all in need of pardon, so that by forgiving others, we can expect some kind of reciprocity, that is, we forgive others with the hope that others in turn will forgive us for our failures. But if we are all bad, there is no guarantee of reciprocity.

I find this line of thought baffling, but of course authors that ascribe this view to Kant develop the point in more detail. Garrard and McNaughton’s remarks about Kant appear in the context of arguing that ‘human solidarity’ provides a reason for unconditional forgiveness, understood as an attitude of love and good will towards wrongdoers who have not necessarily repented. Human solidarity is understood as “the concern for the well-being of those who one feels are in the same condition as oneself” (p. 55). The authors claim that “often it is true to say that in their circumstances we too would have acted as they did” and “even if I could not, as I now am, do what the offender did, nonetheless had my early (and ongoing) circumstances been less favourable, I might have become the kind of person who could act in this way” (p. 54). The idea is that we are not so different to offenders, since we share ‘membership in the human community’ in the sense of a shared common psychology and moral predicament which includes “the possession of [a] morally tainted nature” (p. 54). In a footnote they claim that Kant advocates a similar approach by appealing to “moral luck, as well as the difficulty of knowing the inner springs of motivation, in recommending an attitude of humility rather than superiority to manifest wrongdoers” (footnote, 19, p. 55).

The claim that we all share membership in the human community or the same human nature is surely uncontroversial. The problem is to establish exactly what follows from this. The argument seems to depend on the claim that “often it is true to say that in their circumstances we too would have acted as they did” or at least we would have performed “some similarly awful deed” had our “early circumstances been less favourable.” But this further claim is not self-evident, and nor is it uncontroversial. Thus, considerable further argument is required to establish this point, but Garrard and McNaughton do not provide it. First, it is not clear why ‘less favourable circumstances’ should be linked with the disposition to perform awful deeds. This presupposes certain views about wrongdoing that again are never spelled out by the authors. For Kant, wrongdoing is in broad terms a tendency to either act on subjectively valid motives while recognising that they do not provide justification for one’s actions (weakness) or, more seriously, a tendency to take subjective valid motives as having more objective force than they really have in the sense of being justified from the standpoint of others.^10^ Someone who has had a very ‘favourable’ upbringing (at least in the sense of ‘privileged’) might be particularly prone to thinking that their motives have more objective force than they really have. If by ‘less favourable circumstances’ they count any circumstance that is not favourable to morality, then this seems like an empty claim and perhaps even a circular argument. If what they have in mind is a more substantive account of ‘less favourable circumstances,’ then they should at least explain what those less favourable circumstances are supposed to be and how are they linked to wrongdoing. Second, and more importantly, this line of thought seems to be incompatible with Kant’s theories of freedom, agency, and responsibility. For Kant moral responsibility requires authorship, that is, for Kant, both an agent’s act and his moral character are imputable and this, in turn, implies that for Kant we are *free* to choose our maxims and, as we will see, our characters (Kant 1998, 44).^11^ On the Kantian account, whether or not an act counts as morally wrong in some circumstances would depend on the maxim on which it is performed. The point of an ethics of principles is that what we do is not determined by our circumstances but by the maxims that we *freely* adopt. Thus, from a Kantian perspective, different agents would act differently even in the same circumstances, provided that they have adopted different principles. Finally, the claim that we all share the same common nature and a frail moral predicament is compatible with the view that some agents are virtuous while others are vicious. In fact, the three most important traditions in Western moral thought (virtue ethics, utilitarianism, and Kantianism) share the assumption that the space of human nature allows for a distinction between a virtuous and a vicious character. There is a common human nature, but within the scope of this common nature, there is space for the possibility of cultivating a virtuous character and a moral point of view. The claim that what we do is ultimately determined by our circumstances is a form of situationism, which is clearly alien to Kant. Whatever the merits of Garrard and McNaughton’s approach, we can safely conclude that it is not the one advocated by Kant.

Murphy (1988) reads the passage (Kant 1991, 460–1) as claiming that hatred is never justified (p. 98) and as recommending an attitude of humility rather than superiority to wrongdoers. He interprets Kant as maintaining an unconditional duty to forgive that is grounded on two arguments. First, we cannot know other people’s maxims through an observation of their external behaviour (Kant 1998, 20), so it is for God “who ‘knows the heart’” to decide if “another is evil to the degree that hatred of him would be justified” (1988, p. 98). Second, “each human being is himself so morally flawed as to lack proper standing to hate and despise other human beings” (p. 99), because even those who consider themselves to be good might in fact have avoided vice simply due to lucky circumstances (Kant 1998, 38).

First, it should be noted that the issue of whether Kant’s moral system can accommodate moral luck is itself controversial.^12^ I read Kant’s moral philosophy as allowing one type of moral luck. However, this is not the type of moral luck required by Murphy’s reading. In fact, in the passage of the *Religion* that Murphy quotes in support of his reading (Kant 1998, 38), the point that Kant makes is that there are cases in which people act without consulting the moral law, but have “luckily slipped by the evil consequences” (Kant 1998, 38/p. 30), or cases whether the credit for avoiding vicious acts should “perhaps [go] to good luck” (ibid.). This is just moral luck with respect to whether we would ever face circumstances that would make manifest a fundamental, and hence deeply embedded, evil maxim. Kant can allow for such types of luck (i.e. fundamentally evil people that avoid the circumstances that would make manifest a bad maxim),^13^ but he cannot allow moral luck with respect to whether the fundamental maxim itself is good or evil because for Kant we are morally responsible for our actions and our character, which means that these are freely adopted (Kant 1998, 44). There is no luck with respect to whether agents have a good or evil character, but (perhaps paradoxically) there is some scope for luck for cases in which the agent has an evil fundamental maxim, but due to lucky circumstances, he never in fact performs any external seriously morally bad act (e.g. he would have killed had he been offered a great sum of money, but he never received the offer). In order to ascribe to Kant the (in my opinion bad) argument that we should all forgive each other because we are all evil (Murphy 1988, pp. 99–101), Murphy needs the stronger thesis about moral luck in the choice of our fundamental maxim, but Kant is only committed to the weaker claim that there is luck with respect to whether or not the fundamentally evil disposition is ever externally manifested.

On the problem of *knowing* a person’s true maxim, it is true that Kant insists that we can never be sure of other people’s or even our own motives. The point is an epistemological one, but again we should be careful in assessing what follows from this. We should be cautious in drawing strong moral implications form Kant’s remarks about the epistemological problems stemming from the lack of transparency of our motivations. After all, Kant also claims that our first moral duty is to know ourselves (Kant 1991, 441) and in fact, as we will see, Kantian ethics is an ethics of self-knowledge, which recommends that we embark on a project of self-reform. With respect to our own motivations, we have a duty to strive to improve our maxims even if we can never be sure of their true content. With respect to the motivations of others, as Murphy himself notes (1988, p. 99), even if we can never be sure about other people’s underlying maxims, we are surely able to form reasonable beliefs about them that are based on the available evidence. In many cases, certain external behaviours (e.g. torture of the innocent and various forms of extreme cruelty seem like obvious examples) would almost certainly be indicators of a corrupt character, and from the victim’s point of view if someone has hurt and wronged us surely we are entitled to at least prima facie assume that the maxim of the wrongdoer is morally dubious, at least in the lack of some evidence to the contrary. Kant’s epistemological caution about the possibility of knowing our own and other people’s motives does not ground a general duty to take a forgiving attitude towards wrongdoers unconditionally. Instead, it recommends that we only take a forgiving attitude towards others when we have reason to believe that there is evidence of commitment to a project of moral self-improvement.^14^ Thus, Murphy’s account does not succeed in providing an accurate representation of a recognizable Kantian theory of forgiveness.

## Section IV

I will now argue that on the Kantian account we have a duty to adopt a maxim of forgiving repentant wrongdoers who have embarked on a project of self-reflection and self-reform. The duty derives from the formula of humanity and some considerations grounded on Kant’s theory of rational agency, the thesis of radical evil, and his theory of moral development. This reconstruction appeals to different strands of Kant’s philosophy and goes beyond the cryptic remarks found in the passage under consideration (Kant 1991, 460-1). However, I believe that the argument is compatible with a plausible reading of the passage and Kant’s views on freedom, agency, and responsibility.

In the *Groundwork*, Kant tells us that “[e]verything in nature works in accordance with laws. Only a rational being has the capacity to *act in accordance with the representation* of laws” (Kant 1997, 412/p. 24). The capacity to act under the representation of laws is then equated to the capacity to act “in accordance with principles” and having “a *will*” which is in turn equated to “practical reason” (Kant 1997, 412/p. 24). For Kant the will is practical reason, that is, a faculty of acting through the conception of a principle. Kant distinguishes two types of principles. Objective principles hold for all rational beings and instruct us how we ought to act, and for finite beings like ourselves take the form of imperatives (categorical and hypothetical) (Kant 1997, 413). Subjective principles are maxims, that is, self-given principles of action that hold only for the subject (Kant 1997, 422). For human agents, who have imperfect wills, acting under the ‘representation of laws’ involves acting on subjective principles, and insofar as they are acting rationally, under the command of imperatives. A person’s maxim typically expresses the reasons that motivate her to act as she does. A maxim should be understood as a principle that connects some generic description of circumstances (taken broadly to include the inclinations and purposes of the agent) with some generic description of an action type that the agent takes these circumstances to warrant. Crucially, then, maxims are subjective principles of justification. On Kant’s theory of rational agency, agents act on maxims, which are principles of action that generate, explain and justify external behaviour. The adoption of maxims does not does necessarily or always require an agent’s conscious decision.^15^ As noted, Kant claims that we are sometimes uncertain of our own motivation (Kant 1997, 407; Kant 1998, 20), which means that we are not always explicitly or consciously aware of the maxims that we adopt. Maxims can be adopted tacitly, implicitly and, in many cases, retroactively. However, as maxims are a product of our freedom and principles, for which we are responsible, we can and should become aware of them through reflection (Korsgaard 1996b). The important point is that rational actions have an implicit claim to justification in the sense that the agent takes the circumstances to warrant the acts. Kant can allow for cases of weakness of the will (‘frailty’), in which maxims are adopted only as justifying reasons but fail to motivate (Kant 1998, 29), but these would count as cases of irrationality. Thus, Kantian ethics is an ethics of principles that recommends self-reflection and self-reform and commands that we strive to know ourselves “in terms of [our] moral perfection in relation to [our] duty” (Kant 1991, 441/p. 236) by becoming aware of our maxims and attempting to get rid of those that on reflection we do not fully endorse.

Kant also claims that agents are responsible for their actions and character (Kant 1998, 44), which means that maxims are freely adopted at least in the sense of involving freedom of choice (*Willkür*). Actions are performed freely on the basis of reasons and are not determined by antecedent psychological forces. According to the IT “the will cannot be determined to action through any incentive *except so far as the human being has incorporated it into his maxim*” (Kant 1998, 24/p. 49). This means that incentives never determine the will directly—by exerting a force on the will—but do so through a choice made by the agent that is expressed in the adoption of a maxim. Kant distinguishes two types of incentives: empirical incentives (taken broadly to include inclinations, feelings, and emotions) and the rational incentive of duty, which Kant terms ‘respect for the moral law’ (Kant 1997, 400; Kant 2002, 76). Although both types of incentives might have an affective aspect, they should not be taken as ‘causes’ or ‘pushes’ that directly determine the will, because that would be incompatible with practical freedom in Kant’s sense. Instead the agent must endorse the empirical or rational incentive by “incorporating it into his maxim” and taking it as a sufficient reason for his actions, i.e. as part of the circumstances that warrant the act.

In Kant’s later writings it also becomes clear that maxims can have different levels of generality, implying that agents act not only under maxims but also under a system of maxims that form a hierarchy, with the more particular maxims fitting under the more general ones. Matthew Caswell (2006) has provided a good example of how an agent’s action can be explained by appealing to a system of maxims that form a hierarchy: “Take, for example, my behaviour in laying shingles on a roof. My maxim might run, ‘When making a wood-construction roof, I will nail shingles onto it, in order to build a well-protected covering for my house.’ This maxim fits under the more general maxim, ‘I will build a well-made roof, when constructing my house.’ This in turn might fit under the more general maxim, ‘In order to secure shelter, I will, if possible, build my own house;’ and again, ‘In order to survive the up-coming winter, I will obtain shelter’” (pp. 193–4.) Caswell notes two things. First, higher-order maxims do not fully determine the lower-order maxims that fall under them. The only constraint that the more general maxims impose on the lower subordinate maxims is that they must be a means to the end that the agent has selected. Second, higher-order maxims rationally justify lower-order maxims, that is, it is the whole system of maxims that provides the justification for the agent’s actions. In order to avoid regress, there must be a point where the chain of maxims ends. Kant is explicit about the need for an ultimate principle: “One cannot, however, go on asking what, in a human being, might be the subjective ground of the adoption of this maxim rather than its opposite. For if this ground were ultimately no longer itself a maxim, but merely a natural impulse, the entire exercise of freedom could be traced back to a determination through natural causes- and this would contradict freedom” (Kant 1998, 21/p. 47). Thus, in order to solve the problem of an infinite regress in the chain of maxims, Kant proposes that there is an ultimate, most general maxim, which is itself a product of free practical reason. Thus an agent’s character, her *Gesinnung* or fundamental moral disposition, is itself a higher-order maxim that underlies an agent’s choice of more particular maxims. It is the maxim not of this or that project or course of action, but of a person’s entire life (see Allison 1990, pp. 136–145; Caswell 2006, pp. 191–6). Furthermore, Kant’s ethical ‘rigorism’ entails that every action and morally responsible agent must be characterised as either good or evil, excluding the possibility of a middle term, i.e. cases of actions or people characterised as not entirely good or evil (Kant 1998, 23–4/pp. 48–9).^16^ Kant claims that empirical incentives and the rational incentive of respect for the moral law constitute part of the content of the will of any finite rational being. On the one hand, empirical incentives are all subsumed under what Kant terms the principles of self-love or happiness. The end, happiness, consists in pursuing overall satisfaction in life (Kant 1997, 399), a natural necessary end that we cannot ignore (Kant 1997, 415). On the other hand, consciousness of the moral law is for Kant the most basic ‘fact of reason’ (Kant 2002, 29–50) and thus we are also incapable of completly ignoring the commands of the moral law*.* The moral law is an incentive to moral conduct, which means that for human agents, recognition of the moral character of an action is always an attractive feature of that action, that is, something that makes the action *prima facie* worth pursuing.^17^ Therefore, considered materially, an evil and a good will have the same content, so the difference between a good *Gesinnung* and an evil one must lie in the form of the will, or in the manner in which the contents are combined, that is, in how the two incentives are subordinated, namely which one is incorporated as the condition of the other (Kant 1998, 36). The person with a good character is the person whose fundamental maxim is to make the moral law the supreme condition of all acts, thus subordinating the demands of happiness to the demands of morality (Kant 1998, 36). In the case of a fundamentally good maxim, the moral law functions as the supreme principle of justification of all acts and the agent strives to act only on those maxims that can be fully justified to others (or that treat others’ humanity as an end in itself). In contrast, an evil^18^ person is committed to the promotion of her own happiness unconditionally and complies with moral requirements only insofar as they do not demand a great sacrifice of her own happiness. Evil is understood as a form of irrationality that involves either acting on subjectively valid motives while recognising that they do not provide justification for one’s actions, i.e. they lack objective validity [a form of moral weakness, ‘fraility,’ the first degree of radical evil (Kant 1998, 29/p. 53)], or more seriously, taking one’s subjectively valid motives as having objective validity [the third degree of evil which Kant terms ‘depravity’ (Kant 1998, 30/p. 54)], i.e. as reasons for action to which others ought to defer.^19^

In the *Religion* Kant states that “the human being is by nature evil” (Kant 1998, 32/p. 55). Given Kant’s rigorism, this is usually taken to mean that the *default* or natural position of the human will is in fact evil. This is the so-called thesis of ‘radical evil,’ considered by some as one of the most controversial and difficult aspects of Kant’s moral psychology.^20^ To provide a full account of this thesis and the various problems of interpretation that arise in relation to it is beyond the scope of this article. I will emphasise those aspects that are relevant for my argument. Kant claims that we have a ‘propensity’ (*Hang*) to radical evil, and although the concept of *Hang* is not identical to the concept of *Gesinnung*, some commentators interpret them as both referring to different aspects of the fundamental maxim of an agent (Caswell 2006, p. 199; Allison 1990, p. 153). According to this line of interpretation, *Gesinnung* refers to an agent’s fundamental moral disposition or character, while *Hang* is the free tendency of the will (*Willkür*) to choose in a certain way, i.e. in the case of an evil propensity, the tendency of *Willkür* to give undue weight to non-moral incentives, which implies the adoption of a fundamental evil maxim. This choice is deemed radical and evil because the agent freely chooses to turn away from the moral law, which is always an incentive to morality (Kant 2002, 72), and by doing so he is actively resisting its commands. Although this choice is said to be free (Kant 1998, 44), to the extent that it is also supposed to be universal, Kant says that the propensity to evil is an aspect of human nature, that is, is the human species as a whole that chooses a fundamentally evil maxim (Kant 1998, 32). This universality of the propensity raises serious difficulties because Kant offers no formal proof to back up this claim, appealing instead to the obvious and widespread empirical evidence of wrongdoing in the world (Kant 1998, 33). But empirical evidence is not sufficient to ground a claim of universality, and commentators have felt that a formal proof is in fact necessary.^21^ Despite these difficulties, it is clear that at least in the *Religion* Kant is committed to the universal ascription of a human evil disposition. Kant also says that it is ethically necessary, and therefore it must be possible, to overcome radical evil (Kant 1998, 66–67). To overturn evil is to take on the task of becoming virtuous in the sense of acquiring a good *Gesinnung*: to make one’s commitment to the moral law unconditional. In fact Kant is clear that the basic human struggle is the struggle of overturning evil and attempting to change one’s fundamental maxim. This requires a ‘revolution of the heart’ (Kant 1998, 47, 51), which involves changing the order of subordination of our incentives, making the pursuit of happiness conditional on the demands of the moral law. That is, it is ethically necessary for us to struggle against this evil disposition or propensity (Kant 1998, 66–7).

I will now suggest that the revolution of the heart that is required to overturn evil is a necessary aspect of the moral development of a person. In the second *Critique*, Kant characterised moral development as requiring a gradual process of moral change (Kant 2002, 159–160). However, in the *Metaphysics of Morals*, a later work, written after the *Religion*, in addition to the need for a gradual change (Kant 1991, 477), Kant also refers to the need for a singular moral decision to break away from vice (Kant 1991, 477). Some authors have suggested that this singular moral transformation should be identified with the revolution of the heart, proposed by Kant as a solution to the problem of overturning evil in the *Religion* (Drogalis 2013, p. 3–4; Kant 1991, Intro. p. 18)*.* I would like to further suggest that the revolution of the heart plays a central role in Kant’s theory of moral improvement. It is a necessary condition for the possibility of acquiring virtue understood as the strength to overcome obstacles (vices) and make duty the sole incentive of right acts. Some commentators claim that possession of a fundamentally good maxim is a necessary condition for the possibility of acting from duty and thus for the action acquiring moral worth (Allison 1990, pp. 116 and 119; Timmermann 2009, fn 11, p. 49; Drogalis 2013, p. 18 and ff. and p. 54). Against this view, elsewhere I have argued that a person with an evil *Gesinnung* could on occasion act from duty and that in such cases we should ascribe moral worth to her actions. Goodness of *Gesinnung*, on my reading, is required for the ascription of virtue but not for the possibility of acting from duty and ascribing moral worth to actions (see Satne 2013b). Virtue is the “moral strength of a *man’*s will in fulfilling his *duty*” (Kant 1991, 405/p. 206), and as such it involves a firm resolution to act from duty, no matter how strong the temptation to act wrongly. A person with a good fundamental maxim is virtuous in the sense that she will perform morally good actions reliably. Virtue is the highest achievable level of moral perfection for a human being. The revolution of the heart is a necessary condition for the possibility of a person becoming virtuous and thus ultimately a necessary aspect in her moral development. The revolution does not make moral action possible: a bad person could on occasion act dutifully, because dutiful actions are performed for their own sake, and do not require justification by a meta-maxim (Caswell 2006, p. 205). However, there are two main reasons why the revolution is necessary aspect of the moral development of a person. First, as explained above, the revolution makes possible the acquisition of a virtuous character, that is, reliability of motivation can only be accomplished through the acquisition of a good fundamental maxim. Second, the revolution provides the rational framework that allows a person to abandon her immoral maxims. This is because lower-order maxims are rationally justified by higher-order maxims, so a fundamentally good person has no grounds of justification for more particular immoral maxims. Some commentators have maintained that the revolution of the heart requires divine assistance (Michalson 1989) but there is in fact some clear textual evidence to support the claim that the revolution is a real human possibility: “this change of heart must itself be possible because it is a duty” (Kant 1998, 67/p. 84; see also Kant 1998, 50). The reorientation of one’s will requires a single revolutionary act, but after (or during) the revolution there is still progress to be made (Kant 1998, 47–48; see also 66–67). The striving towards virtue requires constant (endless) progress and a continued effort to approximate an (unattainable) ideal of holiness (Kant 1991, 409; see also Kant 1991, 390), understood as the aim of acquiring a fully reliable and pure form of moral motivation. A good *Gesinnung* provides the framework that allows a person to embark on “the road of endless progress toward holiness” (Kant 1998, 47/p. 67) by making possible the task of abandoning immoral maxims—which ultimately is the main task of a project of moral self-improvement.

The concept of a revolution of the heart, however, also presents some difficulties.^22^ On the one hand Kant’s language of change, transformation, and even ‘rebirth’ (Kant 1998, 47) suggests that the revolution has a temporal dimension, yet Kant says that the choice of evil *Gesinnung* is an ‘intelligible deed’ and does not occur in time (Kant 1998, 31), which some commentators interpret as implying that the choice of a good *Gesinnung* is equally timeless (Allison 1990, p. 154). Although it makes sense to think that the revolution does not occur at a precise point in time, it is difficult not to think of it as occurring in time, at least in the sense that it happens to a person, during the course of her life. Perhaps the point about temporality relates to the fact that moral revolutions occur through a free choice (Kant 1998, 51), and as such they require an act of noumenal volition that cannot be temporally located. The alleged timelessness of the revolution could create a problem for the argument that I am developing here.^23^ It is difficult to see how a revolutionary volitional act occurring outside time could be part of the moral development of a person, particularly since the idea of development seems to imply a progression of different chronological stages. There have been different attempts to understand the relationship between the timeless revolution and the gradual process of moral-self-improvement involved in the ethical project of self-knowledge and self-reform. Some authors argue that the revolution of the heart is timeless in the sense that the process of overcoming radical evil occurs simultaneously with the process of improving the morality of one’s maxims, a process that takes place over the course of a person’s life (Sussman 2005b p. 173; Korsgaard 1996a, pp. 180–1). In contrast, Drogalis (2013) has suggested that although the choice of *Gesinnung* does not itself occur in time, “there is a clear relationship between this noumenal choice and that which occurs in time” (p. 145) arguing that “the choice to undergo a revolution can be impacted—though not determined—by empirical activities and should not be viewed as unable to be placed at a moment of a person’s life” (p. 166). I do not wish to enter into this debate here, since I think that my argument about conditional forgiveness is compatible with both readings. The argument only requires that we accept that Kant is committed to the claim that the revolution of the heart is a necessary condition (or aspect) of the moral development of a person. Moreover, some interpreters have suggested that Kant’s characterization of the choice of *Gesinnung* as timeless and intelligible is not particularly problematic. Caswell (2006), for example, notes that “the characterization of the … *Gesinnung* as timeless and intelligible is actually just the application of the theory of the maxim.” Since maxims are just reasons for action, they are intelligible grounds that figure implicitly in the justification of our actions and “we need not make ourselves explicitly aware of our reasons when acting” (p. 200). The concept of political revolution can help us to understand the type of rational transformation that is required. When a political revolution takes place, what was legitimate in the old political regime becomes illegitimate in the new post-revolutionary order. A revolution implies a change in the principles of political legitimacy in a particular society. Analogously, a change of meta-maxim implies a change in the ultimate principle of justification of a person’s will. The meta-maxim structures and shapes a person’s will, so an action that could have been taken as justified under the old fundamental maxim would not necessarily be justified under the new one. Typically, a person who has undergone a revolution of the heart would come to see some of her old maxims as now being unjustified. And as noted above, even after (or perhaps simultaneously with) the revolution, there is more progress to be made (Kant 1998, 47–8) because although the revolution rules out ‘vice,’ that is, the principle of deliberately violating a duty (Kant 1991, 380), it does not rule out ‘impurity’ or ‘fraility’ (Kant 1991, 408). After (or during) the revolution, the agent should still revise her maxims in order to make sure that moral actions are performed out of a pure sense of duty, and will need to continue cultivating a firm resolution of the will in order to live up to her new maxims. The revolution does not imply a transition to actual holiness but a firm resolution to struggle to commit unconditionally to the moral law. Moral development, thus, involves an on-going and self-imposed intellectual process of self-knowledge, reflection, and self-reform.

The next step in the argument is to note that abandoning our immoral maxims would necessarily involve repentance^24^ for our immoral acts. Although not all our immoral maxims are other-directed—Kant thinks that we also have duties towards ourselves—, it is clear that in many cases this process would require that we repent wrong acts committed against others. Repentance here is understood in fairly minimal terms as the commitment to abandon immoral maxims and become a better person. It might involve guilt, remorse, and other forms of painful regret, but not necessarily. 25 What is necessary is that the agent comes to see the maxims underlying her immoral acts as something that cannot be fully justified to others, and makes a commitment to change those maxims. From the point of view of a person who is involved in a process of moral self-reform, the judgement that her maxim is unjustified and the realization that she has wronged others would necessarily involve repentance and in many cases would also involve taking steps (e.g. apology, compensation, and penitence, among others) towards the reparation of the wrong that she has committed. Repentance is thus a necessary aspect of moral development of a person.

With these considerations in place, in the next section I will be able to complete the argument and derive the conditional duty to be forgiving from Kant’s Formula of Humanity (FH).

## Section V

The considerations developed in the previous sections suggest that Kantian ethics recommends that we adopt forgiving attitudes (and practices) towards wrongdoers that have repented their immoral maxims as part of a project of moral self-improvement. However, since Kantian ethics takes the Categorical Imperative (in all its formulations) as the supreme principle of all actions, the argument would not be complete if we could not show that this conditional duty can also be derived from the Categorical Imperative. I have chosen to focus on FH because this is the formula that Kant himself uses most often when deriving particular duties in the *Metaphysics of Morals* (Wood 1999, p. 139).

FH says: “So act that you use humanity, whether in your own person or in the person of any other, always at the same time as an end, never merely as a means” (Kant 1997, 429/p. 38). Kant also tells us that the Categorical Imperative is grounded on the recognition that “*rational nature exists as an end in itself*” (Kant 1997, 427, p. 37). Human persons (and rational beings in general) possess rational nature and it is in virtue of their possessing this nature that persons have a value that requires that they should always be treated as ends in themselves (Velleman 2008). Ordinary ends are reasons or purposes for action. Every day ordinary ends are usually contingent on agents’ desires and preferences and may vary from person to person. Contingent ends are thus rationally optional and conditional on the agent’s set of desires and preferences. In contrast, an end in itself necessarily provides a compelling reason for every agent to act in certain ways. Ends in themselves provide reasons or considerations that we cannot rationally ignore. In particular, to treat something (or someone) as end in itself is to avoid treating it (or them) as a means for the satisfaction of some contingent desire-based ends. For Kant, then, rational nature is an end in itself and in virtue of possessing such nature persons possess an absolute worth or intrinsic value. In virtue of this absolute worth or dignity, persons are worthy of ‘respect.’ Thus FH is usually understood as commanding respect for persons, i.e. to treat their rational natures as ends in themselves.

The correct interpretation of FH is of course open to debate. 26 One important issue is how to understand the scope of ‘rational nature.’ It is generally agreed that Kant is referring to practical reason, not theoretical reason here. But practical reason for Kant is a complex capacity. Practical reason has to do with the exercise of our will and involves the power to make choices about what ends we will adopt (*Willkür*), a power that a rational being should exercise in accordance with instrumental and prudential requirements as well as in accordance with the moral categorical commands of the ‘legislative’ part of the will (*Wille*). On some readings, the end in itself just encompasses humanity understood as the power to set ends (*Willkür*) (Korsgaard 1996a, p. 17, p. 110, p. 346; Wood 1999, pp. 118–120). However, there are some texts suggesting that for Kant it is morality, or more specifically the capacity for morality, that provides the distinguishing feature of beings that are ends in themselves: “morality is the condition under which alone a rational being can be an end in itself.… Hence morality, and humanity insofar as it is *capable of morality*, is that which alone has dignity” (Kant 1997, 435/p. 42, my emphasis).^27^ Thus, according to one influential and plausible reading, it is the ‘capacity for morality’ that is an end in itself.^28^ The ‘capacity for morality,’ which is an end in itself and the source of the respect owed to persons, includes the capacity to set ends (*Willkür*) plus the capacity to legislate moral principles (*Wille*) and the capacity to act in accordance with those principles, i.e. the capacity for rational self-constraint that Kant terms “respect for the law” (Kant 2002, 76, p. 99)^29^

Some authors have argued that the Kantian notion of respect actually provides considerations in support of unconditional forgiveness of wrongdoers, that is, irrespectively of whether or not they have repented. Margaret Holmgren (1993) has appealed to “a Kantian conception of the intrinsic value of persons” (p. 349) in order to argue that respect for persons requires that we be prepared to forgive wrongdoers unconditionally. She notes that for Kant we are autonomous beings capable of rational choice, which means that “we have the *capacity* for a good will” (p. 349, my emphasis). This aspect of human nature marks us out as morally responsible agents and “imparts to us our intrinsic value” (p. 349). She also notes that this intrinsic worth is not “defeated by our wrong choices or mistaken attitudes,” that is, respect is owed to every person as an end in herself, that is, in virtue of her intrinsic worth and irrespectively of whether or not she possesses a morally bad character or has performed some wrongful deeds. Although Holmgren’s article does not engage with a close analysis of Kant’s texts, her characterisation of the Kantian notion of respect is broadly accurate and in line with the interpretation of FH that we have adopted here. However, Holmgren also maintains that respect for someone who has wronged us requires that we be prepared to forgive her while fully recognising and condemning the act as wrong, because she claims that respect is incompatible with “resentment, hatred and ill will” (p. 349). On her view, our capacity for moral agency and rational change is an object of respect, and respect in turn requires that we be prepared to forgive wrongdoers irrespectively of whether or not they have chosen to exercise these capacities by repenting their immoral acts.^30^

Holmgren is correct in pointing out that the mere capacity for morality and autonomy warrants respect, and that respect is owed to all persons, including wrongdoers, but she is mistaken in claiming that respect necessarily requires unconditional forgiveness. In fact, one can refuse to forgive an unrepentant wrongdoer without disrespecting her.^31^ Respecting a person’s capacity for morality and rational choice does not necessarily require forgiveness because a person can exercise her capacity for rational choice to choose an evil fundamental maxim. This choice can be deemed ‘evil’ precisely because the moral law is always an incentive to morality, that is, precisely because choosing to commit unconditionally to the moral law is always a ‘live’ option for a person. Moreover, resentment, understood as a self-regarding form of anger caused by having been injured or harmed by a morally responsible agent (Hieronymi 2001), is a warranted response to wrongdoing *precisely* because the wrongdoer is seen as a free agent who should and could have acted otherwise. If the wrongdoer chooses to endorse his original immoral maxim by remaining unrepentant for his immoral deed, then it seems that the victim’s reasons for resentment are compounded rather than dissipated. In fact, in this kind of case, respect for persons provides justification for the victim’s refusal to forgive the offender.^32^ Thus, contrary to Holmgren, respect and recognition of a person’s humanity, understood as a capacity for moral rational choice, would sometimes not only be compatible with resentment and other negative attitudes towards the offender, but actually require it.

So far I have shown that respect does not necessarily require forgiveness, that is, the duty to forgive offenders is not unconditional. I now want to show that FH can ground a duty of virtue to adopt a maxim of cultivating forgiving attitudes (and practices) towards wrongdoers who have repented as part of a commitment to a project of moral self-development. Holmgren’s mistake, in my view, stems from a failure to fully acknowledge the role that Kant’s theory of radical evil should play in a Kantian conception of forgiveness on the one hand, and a failure to recognise the Kantian duty to be forgiving is wide and imperfect, on the other hand. Thus, we need to explain the nature of these duties and how they are derived from FH.^33^ The duty to be forgiving is one of the duties of sympathy, which, together with duties of beneficence and gratitude, are part of the duties of love that we have to others (Kant 1991, 448–462). Duties of love are duties of virtue that have as their objects obligatory ends, i.e. “an end that is also a duty” to have (Kant 1991, 383). Duties of virtue are, thus, primarily duties to have certain ends and to adopt, correspondingly, certain maxims. Moreover, duties of love are wide or meritorious: the specific actions are not strictly owed but the agent still deserves moral merit for performing them. FH establishes that there are certain obligatory ends, i.e. my own perfection and the happiness of others (G 430). These ends are obligatory because their adoption is required if we are to fully respect others and our own rational natures. We respect our rational nature by cultivating the capacities that are necessary for achieving all sort of ends and respect the rational natures of other people when we help them to pursue various ends that are part of their rational conception of happiness (Wood 1999, p. 149). In the case of duties of love, the obligation to comply with these duties stems from the fact that promoting the happiness of others is an obligatory end (Kant 1991, 385). A duty is a duty of love if the action promotes a duty of virtue, that is, an end that it is a duty to adopt. So, for example, a particular act of charity might count as a duty of love insofar as it promotes the happiness of others. Now, the important thing to note is that the duty to promote an end does not require that we attempt to promote the end in every possible opportunity, i.e. that we should attempt to maximize the happiness of others as much as we possibly can.^34^ These duties are imperfect and that means that there is some latitude for agents to decide in what way (through what specific actions) and to what extent (how far) to promote these ends (Kant 1991, 390–4). Their latitude stems from the fact that duties of virtue do not command us to act in specific ways, but rather to adopt certain principles. The duty to promote an end is, thus, primarily a duty to refrain from adopting the maxim of refusing in principle to promote that end (Wood 2009, p. 234). So, for example, in the case of the duty of beneficence, Kantian ethics forbids that we adopt a maxim of never helping others to achieve their ends (Kant 1991, 452).^35^ This is a very important point to stress in relation to the duty to be forgiving: Kantian ethics forbids that we adopt a maxim of refusing in principle to develop forgiving attitudes towards wrongdoers. Thus, for Kant, there is no unconditional duty to forgive all persons that have wronged us directly or indirectly. Instead what Kantian ethics rejects is a maxim of unconditional unforgivingness, that is, what is impermissible is to adopt a maxim of never forgiving anyone.^36^ As there is latitude^37^ in deciding what and how much to do in relation to the duty of beneficence, there is also some latitude to decide in what way and when to forgive wrongdoers. Duties of sympathy recommend that we ‘cultivate’ certain sympathetic attitudes and compassionate feelings “as means to promoting active and rational benevolence” (Kant 1991, 456/p. 250). Relatedly, we also have duties to overcome the vices of envy, ingratitude, and malice, which tend to be obstacles to the development of our sympathetic attitudes. In section II we noted that, according to Kant’s Incorporation Thesis, empirical incentives like inclinations, emotions, and feelings influence the will by being incorporated into maxims: agents act on empirical incentives by either endorsing or refusing to endorse them in their maxims. Since empirical incentives cannot be generated at will, a duty to ‘cultivate’ certain emotions and feelings is a duty to critically evaluate these feelings in light of moral principles and to use practices of self-examination and reflection to attempt to eradicate (or at least refuse to endorse) those tendencies that are deemed morally inappropriate.^38^ In the case of the duty to be forgiving, what we should do is to adopt a maxim of cultivating forgiving attitudes (and in some cases other possible forgiving practices) by critically evaluating certain negative emotions that are typically felt towards wrongdoers and refusing to endorse those that on reflection we deem morally inappropriate. To adopt a forgiving maxim is to take on the task of cultivating a forgiving character, i.e. to be prepared to forgive when the circumstances are deemed appropriate.^39^ The question, then, is: what are the circumstances that make forgiveness appropriate?

Duties of virtue command the adoption of maxims, so that the application of these maxims in specific circumstances requires that we “call upon judgment to decide how a maxim is to be applied in particular cases” (Kant 1991, 411/p. 211). Granting this element of practical deliberative judgement and given the arguments developed in section IV, it should be clear that the chief consideration that makes forgiveness appropriate is repentance of immoral acts as constituting evidence of the offender’s commitment to abandoning immoral maxims as part of a project of moral self-improvement. There are various considerations that provide support for this interpretation. First, if the wrongdoer refuses to endorse his original immoral maxim by becoming repentant of his immoral deed, then it seems that the victim would be justified—if not fully at least partially—in overcoming her resentment. It would certainly be the case that her reasons to continue to resent would be weakened and certainly a lot weaker than in cases in which the offender remains unrepentant.^40^ Second, as the wrongdoer has now abandoned the immoral maxim, there is some ground for the hope that she will not act in similar ways in future, and hence some reasons to refuse to hate her and even to consider the possibility of restoring a relationship with her. Third, Kant says that although we have a duty to promote the happiness of others, which includes helping others to achieve or realize some of their ends, it is impermissible to help others to pursue immoral ends (Kant 1991, 450). In fact, as we have seen, the end of happiness is permissible only on the condition of being pursued in accordance with morality. Kant is clear that we do not have a duty to promote immoral happiness (Kant 1991, 388 and Kant 1991, 480–1). He also claims that we cannot make the moral perfection of others a matter of our duty, because this is something that “only the other himself can do” (Kant 1991, 386/p. 191) and clearly respecting other people’s autonomy precludes that we attempt to make their moral perfection our own end. But the duty of moral self-knowledge is the first command of all duties to oneself (Kant 1991, 441), so it would be peculiar if we did not have some duties to others in virtue of their commitment to moral self-perfection. Kant himself recognises that the happiness of others “also includes their *moral well-being*” (Kant 1991, 394, p. 197), claiming that we have at least a negative duty to promote their moral perfection by not tempting them to immorality (Kant 1991, 394). So, although a person’s project of moral development is clearly a deeply personal project that can only stem from her own reason and autonomy, other people’s perfection can be a duty for us at least indirectly, that is, insofar as it belongs to their ends and thus is part of their permissible happiness (see Wood 1999, p. 326). This means that we have an indirect duty to promote the perfection of others at least insofar as perfection is one of their ends that constitute their happiness. These considerations provide support for conditional forgiveness. In addition, there are two further reasons to think that the victim’s forgiveness could somehow help or complete the moral development of the wrongdoer (without compromising either the victim’s or the wrongdoer’s autonomy). Repentance might require ratification by others (Hieronymi 2001 p. 550) because if repentance is always met with indifference it might make it psychologically difficult for the wrongdoer to engage in the required process of moral self-reflection, so that by forgiving others we somehow help them in the process of abandoning immoral maxims.^41^ As noted by Allen Wood, moral progress and the struggle against radical evil “can be effective only if it is carried out through an ethical community” (Wood 1999, p. 332; see also pp. 335–6). So we have a duty to bring about just and fair institutions, that is, institutions that do not encourage radical evil. This would mean on the one hand promoting just institutions that punish enforceable wrongs, but on the other hand, in the personal sphere, it would mean that there is space for conditional forgiveness to play a role in the struggle against radical evil, at least to the extent that forgiveness could, for example, help to restore personal relationships and reintegrate wrongdoers into the community.

Kant also says that we should exercise epistemological caution with respect to other people’s maxims because we can never *know* whether this revolutionary process has taken (or is taking) place (Kant 1998, 45, 47, and 50). Ultimately the forgiver should exercise her judgement in deciding whether or not the wrongdoer’s repentance is sincere, but equally the wrongdoer should be prepared to engage in a project of self-reform even if forgiveness is not forthcoming. It is also important to note that Kant does not classify the duty to be forgiving among the duties of respect. These are also ethical duties, but they are strict or narrow duties, that is, duties that are strictly owed, require specific actions or omissions, and can incur moral culpability (Kant 1991, 462-68). Among these duties, Kant includes the duties to avoid arrogance, defamation, and ridicule. Duties of respect are directly derived from FH and command that we avoid acting in ways that show direct disregard for the worth or dignity of humanity (see Wood 2009). If Kant had thought that the duty to forgive was unconditional and derived directly from a duty to respect the intrinsic worth of rational beings, as argued by Holmgren (1993), he would have included the duty to forgive among the duties of respect to others. Instead he classifies it as one of the duties of love.

We are now in a position to reply to Sussman’s assertion that forgiveness sits uncomfortably in Kant’s system. I have argued that Kant’s ethical system can ground a duty of virtue to adopt a maxim of developing forgiving practices and attitudes towards wrongdoers under circumstances that are deemed appropriate (chiefly repentance as evidence of a commitment to a project of moral self-improvement). It turns out that from a Kantian perspective, forgiveness is not ‘elective’ but, at least in some cases, morally required.^42^ Philosophers are sometimes unsympathetic to the view that we have a duty to forgive (Hallich 2013) in part because the notion of a right to be forgiven seems problematic (Sussman 2005a, p. 87). But we should note that Kantian ethical duties cannot be enforced, because the adoption of ends cannot be compelled (Kant 1991, 381) and, more importantly, because others are not entitled to coerce compliance—because compliance is not owed, i.e. imperfect duties do not correspond to rights (Stratton-Lake 2008, p. 106). The Kantian notion of a duty of virtue can retain the idea that there is a duty to be forgiving while at the same time rejecting the view that wrongdoers have a right to be forgiven.

## Section VI

I have argued that we do not have a duty to forgive those who are not engaged in a project of moral improvement and self-reform. I now want to suggest that Kant goes a step further and maintains that forgiveness of recurrent wrongs would actually involve a violation of a duty to oneself. Towards the end of the passage, Kant says:

But this [the duty to be forgiving] must not be confused with *meek toleration* of wrongs (*mitis iniuriarum patientia*), renunciation of rigorous means (rigorosa) for preventing the recurrence of wrongs by other men; for a man would be throwing away his rights and letting others trample on them, and so would *violate his duty to himself*. (Kant 1991, 461, p. 253 my emphasis)

Here the reference to “meek toleration” of “recurre[nt] … wrongs” strongly suggests that the wrongdoer has not repented, so the passage adds textual support to the view that the duty to forgive is conditional. Moreover, Kant now seems to be introducing a new idea and committing to a stronger claim, that is, the forgiveness of recurrent wrongs (unrepentant wrongdoers) would involve a violation of a duty to oneself. In section II, I noted that if forgiveness is understood as merely refraining from the desire for revenge, then it seems problematic that Kant is recommending that we “hate our enemies out of mere revenge” even in cases whether the wrongdoer has not repented. Kant seems to imply that we are under an obligation to do everything we can to prevent the recurrence of wrongs (within the limits of moral and perhaps legal permissibility). Endorsing a maxim of hating our enemies out of mere revenge does not seem very conducive to the prevention of the recurrence of wrongs by other people. So this passage also provides further support for a broader definition of forgiveness (see section II). In any case, it is clear that now Kant is adding that any form of forgiving that involves meek toleration of wrongs would constitute a violation of a duty to oneself and as such is morally impermissible.

The passage does not clarify what exact duty we would be violating by forgiving unrepentant wrongdoers, but a careful look at Kant’s classification of duties suggest that the “duty to oneself” that Kant refers to here (Sussman 2005a, p. 88) can only be the duty of *self-esteem* (Kant 1991, 435–6), that is, the duty to respect and recognise our own dignity as rational beings, which involves the overcoming of the vice of servility or false humility (Kant 1991, 434–7). This is one of the perfect duties to themselves that human beings have merely as moral beings (Kant 1991, 429–37) and “which involves the preservation of oneself as a moral being” (ibid.). The implication is that certain forms of forgiveness might involve a lack of self-respect and as such display the vice of servility. The idea of a duty not to forgive is easier to understand if we define forgiveness more generally as involving the overcoming of negative emotions and other practices such as the restoration of relationships and reintegration of the wrongdoer into the community. If resentment is understood as a self-regarding form of anger caused by having been injured or harmed by a moral responsible agent, and the object of resentment is understood as the lack of respect shown to us by the wrongdoer, then resentment could be seen as a legitimate form of defending one’s self-respect, and as such not always morally reprehensible. Similarly, restoring a relationship or welcoming a wrongdoer into the community might be impermissible if the wrongdoer has not repented at all and has not embarked on a project of moral transformation and self-reform.

Meek toleration of recurrent wrongs would involve a lack of self-respect because the wrongdoer, *ex hypothesi*, has not repented, so she still endorses the maxim that underlies her wrongful action. Insofar as the wrongdoer has not withdrawn the maxim, the wrongful act still stands as a lack of respect against the victim. Quick forgiveness of disrespectful acts would involve a ratification of the lack of respect embedded in the immoral maxim and as such a failure to recognise and respect our own dignity as rational beings. A failure to recognise our own dignity as a source of unconditional value is a failure to recognise the legitimacy of the moral law as the supreme law of the will, and is it easy to see how this lack of self-esteem could jeopardize both the preservation of oneself as a moral being and ultimately one’s ability to act morally. Forgiveness of the unrepentant might also show disrespect to the wrongdoer, who should be treated as an autonomous moral agent accountable for his acts. It is precisely because I recognise the dignity of the wrongdoer that I resent his acts insofar as the wrong involves a moral injury against me. Repentance, thus, has a double function. On the one hand, the capacity for rational change on the part of autonomous agents might ground a moral transformation that warrants forgiveness. On the other hand, repentance (in most cases) also allows the victim to forgive without compromising her self-respect.

We should also consider whether a conflict of duties could arise in some cases. It should be noted that in cases where there is no repentance, there is no conflict of duties because there is no duty to forgive. Potentially problematic cases would be those in which the wrongdoer has repented, but forgiving would still involve a lack of self-respect for other reasons (for example, cases in which the wrong is very serious, e.g. crimes against humanity or cases whether there are serious aggravating circumstances). There are probably cases in which the duty to be forgiving could conflict with the duty of self-esteem. Whether this is the case is something that the victim needs to judge in each particular case. If conflict occurs, then the duty of self-esteem takes priority because it is a perfect duty (i.e. a duty that we should never violate). The account of the duty not to forgive and the possibility of potential conflict between duties that I am providing here is certainly very sketchy—a more careful exploration of these arguments is a task for future work. In particular, the justification of the duty not to forgive requires more careful consideration. On the one hand, a necessary link between the notions of lack of self-esteem, servility, and forgiveness of unrepentant wrongdoers is not unproblematic: there have been historical cases of persons (Mandela, Gandhi, and Socrates are famous examples) that seem to have managed to forgive unconditionally without compromising their self-respect (Hallich 2013, p. 1011). On the other hand, the connection between quick forgiveness of unrepentant wrongdoers and servility requires more spelling out given Kant’s definition of servility as the “waiving [of] any claim to moral worth in oneself, in the belief that one will thereby acquire a borrowed worth” (Kant 1991, 435, pp. 231) and as “belittling one’s own moral worth merely as a means to acquiring the favor of another” (Kant 1991, 436, pp. 231). as well as the examples that he gives to exemplify this vice (Kant 1991, 435–6).

## Section VII

I have argued that forgiveness does not sit uncomfortably in Kant’s moral theory. On the contrary, I have shown that there is space in Kant’s philosophy for a genuine theory of forgiveness. We have a duty to be forgiving, that is, a duty to adopt a maxim of cultivating forgiving attitudes (and practices) in circumstances that are deemed appropriate (chiefly repentance as evidence of commitment to a project of moral self-improvement). The duty to forgive is a duty to adopt a forgiving maxim towards our fellow human beings (under certain conditions) and, since maxims are principles of justification, forgiveness on this account is paradigmatically responsive to reasons. The reason to forgive others is based on the recognition that the human predicament is a predicament of evil, but precisely for that reason, our first duty is a duty of self-knowledge that commands us to embark on a project of reflection and self-reform through a revolution of the heart. The capacity to comply with this duty stems from our own rational nature and autonomy, and when we have reason to believe that others have also embarked on this project, we should be prepared to take a forgiving attitude towards them. I have also suggested that, for Kant, quick forgiveness of unrepentant wrongdoers is impermissible because it implies a violation of a duty of self-esteem. Some aspects of this account require further investigation and development. In particular the link between forgiveness of unrepentant wrongdoers, lack of self-esteem, and servility requires more spelling out. However, although these topics are important directions for future work, I hope that the argument I have developed here will lay the grounds for the correct interpretation and reconstruction of a recognisable Kantian theory of forgiveness.

## Footnotes

1 Citations of Kant’s work will reference the page number of the Prussian Academy edition. Direct quotes will also provide the page number in translation. Translations used are indicated in the bibliography.

2 In his article, Sussman (2005a) also attempts to develop a “recognizable Kantian account of forgiveness” (p. 85) by appealing to Kant’s views of God’s grace as a kind of moral archetype for the kind of forgiveness available to human beings. I do not discuss Sussman’s proposal because I aim to show that Kant’s central theses already have the necessary resources to develop an account of forgiveness and thus there is no need to appeal to the doctrine of divine grace.

3 I see this suggestion as an improvement on Kant’s definition, but one that is compatible with Kant’s central commitments.

4 The philosophy of emotions has generated a lot of interest and debate in recent decades. For an overview of the different positions, see Deonna and Teroni 2012.

5 For recent discussions of Kant’s conception of emotions, see Cagle (2005); Cohen (2014) and Williamson (2015).

6 For a more detailed examination of the maxim of forgiveness, see section V.

7 Again, here I am improving on Kant’s account but in ways that are fully compatible with his fundamental commitments.

8 Thanks to an anonymous referee for *Philosophia* for pointing out the importance of recognising Robert’s distinction. Although the two accounts are akin to the extent that they recommend ‘forgivingness’ rather than a duty to forgive, there are also important differences between the two. Roberts justifies the need for the virtue of forgiveness by reference to a “definition of a virtue as a trait that fits one to live one’s life well in some distinctively human dimension” (1995, p. 289). Instead Kant appeals to the notion of a wide or imperfect “duty to be forgiving,” which is grounded on Kant’s formula of humanity (see section V). In addition, the conceptual categories used by both authors are very different: Roberts relies on his understanding of emotions as “concerned construals” (see his 1988) and sees the overcoming of anger as a change in the victim’s perception of the wrongdoer in light of reasons characteristic of forgiveness. In contrast, Kant refers to the concept of maxims, not dispositions, and understands the possibility of overcoming negative emotions in terms of incorporating/refusing to incorporate incentives into maxims. The reasons that warrant forgiveness are based on considerations specifically related to Kant’s theory of radical evil and moral development.

9 Here I do not intend to offer a comprehensive overview of the different interpretations available in the literature.

10 See section IV.

11 According to Kant’s theory of rational agency, our character is determined by our choice of fundamental maxim (*Gesinnung*). See section IV.

12 In recent decades there has been a lot of interest and debate surrounding the notion and possibility of moral luck [for the most important positions see Statman (1993)]. The debate was inspired by Williams (1981) and Nagel’s (1979) seminal articles on the topic. Nagel defines moral luck as a situation “where a significant aspect of what someone does depends on factors beyond his control, yet we continue to treat him in that respect as an object of moral judgment” (p. 26). Both Williams and Nagel claim that Kant’s ethics denies the possibility of moral luck (Williams 1981, p. 21; Nagel 1979, p. 26). Yet, the issue is not as straightforward, although most commentators accept that Kant’s theory of moral worth excludes the possibility of moral luck (see for example Allison 1990), some interpreters are happy to admit a degree of moral luck on either Kant’s theory of virtue (Herman 1993) or his theory of grace (Sussman 2005a) or at least an element of luck with respect to temptations that would make manifest a bad fundamental maxim (see Kant 1991, 392, Caswell 2006, and Satne 2013b).

13 See Satne 2013b, pp. 27 and ff.

14 See sections IV and V.

15 See Brewer (2002) and Bittner (2001) for a good discussion of Kant’s views on maxims.

16 For a brief reconstruction of Kant’s argument in support of rigorism, see Satne 2013b, p. 21.

17 This thesis entails a form of motivational internalism that rules out the possibility of human beings being aware of moral obligation with either indifference or contempt (see Satne, 2013a p. 619).

18 Here ‘evil’ just means morally bad.

19 In the second *Critique* this form of wrongdoing is equated with self-conceit (Kant 2002, 73). See Allison 1990, p. 124 and Reath 2006, p. 24.

20 For an overview of some of the main difficulties associated with Kant’s radical evil thesis, see Morgan (2005), pp. 63–65.

21 In the literature there are various competing attempts to reconstruct “the missing formal proof of humanity’s radical evil” (Morgan 2005). See also Caswell (2006); Allison (1990), and Wood (1970 and 1999).

22 See, for example, Loncar (2013) for some of the problems associated with the idea of conversion.

23 This relates to the well-known and more fundamental problem of how to understand the relationship between free moral agency and temporality in Kantian ethics. See Stern (1986); O’Connor (1985), and Korsgaard (1996a).

24 Other authors that argue that forgiveness is morally appropriate when there is repentance are Calhoun 1992; Griswold 2007, and Roberts 1995.

25 Some authors claim that genuine repentance should involve painful remorse (see Gaita 2004 and Fricker 2014). Kant’s account is minimalist because although repentance might include pain, it is not the pain that grounds a conditional duty to forgive but rather the commitment to abandon immoral maxims as part of a project of self-improvement and self-reform.

26 For a good overview of some of the key issues, see Dean (2009).

27 See also Kant 1991, 422–3, Kant 1991, 436, and Kant 1991, 464.

28 Hill (1992), pp. 40–1; Herman (1993), p. 238; O’ Neill (1989), pp. 137–8.

29 See Dean (2009). Dean notes that a mere capacity for morality is not to be rational in the fullest possible sense because full rationality requires that the capacity be exercised or realized, that is, the rational being’s commitment to actually act on moral principles. But if rational nature requires full realization, this would have the unpalatable consequence that respect is only owed to beings that are fundamentally committed to morality. Although Dean has developed some arguments to mitigate this worry (see 2006 and 2009), he himself admits that his reading is not conventional among Kant commentators.

30 Trudy Govier (1999) also appeals to respect for persons as a reason to forgive albeit only conditionally.

31 I thank an anonymous referee from *Philosophia* for prompting me to clarify this point and provide a more detailed analysis of the relationship between respecting and forgiving wrongdoers.

32 I agree with Garrard and McNaughton (2003) when they claim that respect for persons is “a background condition of the possibility of forgiveness *or* the refusal to forgive” (pp. 54 and 53). See also section VI.

33 Incidentally, it is worth noting that in the passage we have been considering (Kant 1991, 460–1), Kant establishes the duty to be forgiving without explicitly appealing to any of the formulas of the Categorical Imperative (Wood 1999, p. 141). Here I am attempting to spell out the derivation that Kant fails to provide.

34 In support of this interpretation see Wood (2009) p. 234 and Baron and Fahmy (2009), p. 219.

35 It also requires that we refrain from making the unhappiness of any person our end (see Wood 2009, p. 234).

36 Although not exactly the same point, at the beginning of the *Critique of Practical Reason* Kant gives the example of the maxim “to endure no affront unavenged” as an example of a maxim that is impermissible and cannot be a practical law (Kant 2002, 19). In a similar vein, the maxim “to never forgive an offender under any circumstance” is impermissible. Yet this does not mean that the maxim “always forgive offences” is obligatory.

37 Latitude is a feature of all duties of wide obligation (Kant 1991, 390), including the duty to promote one’s natural perfection and duties of love to others. It can be difficult to understand how duties that are not duties of beneficence could possess latitude, in particular if it is assumed that the latitude of the duty of beneficence is mainly due to human finitude concerning time and resources (Scarre 2004, p. 55, fn 6). But latitude should not be interpreted in this restrictive way. In fact, Kant says that the duty of beneficence “has in it latitude for doing more or doing less” (Kant 1991, 393/p. 197), and this suggests that the obligation to be charitable is not an obligation to do as much as one can possibly do given one’s resources (Baron and Fahmy 2009, p. 219). Duties of virtue possess latitude mainly because they only command the adoption of maxims of ends and as such they leave unspecified both the precise actions that might promote these ends and precisely how much one is to do in order to promote them (Baron and Fahmy 2009, p. 218 and Wood 2009, p. 234).

38 I follow Fahmy’s characterization of the Kantian notion of ‘cultivation’ (see Fahmy 2009).

39 The duty is a duty to be forgiving rather than a duty to forgive. See section II.

40 I allow a degree of judgment on the victim’s part. Repentance provides a weighty reason in support of forgiving the wrongdoer, but there might be cases in which there are other reasons (e.g. the seriousness or nature of the offence) that might make forgiveness inappropriate.

41 Kant also claims that the revolution can only be fully accomplished if we undergo a ‘moral rebirth,’ which involves wiping out an infinite ‘guilt’ or ‘debt’ (*Schulden*) for our previous evil disposition (Kant 1998, 72, p. 88). Some commentators have interpreted this aspect of Kant’s account as implying that the only way of removing this debt, and hence a necessary condition for the possibility of overturning evil, is divine forgiveness (Adams, 1998, introduction, p. xv). The issue of whether or not we should interpret Kant’s position as implying that we need divine forgiveness in order to fully accomplish our moral rebirth is irrelevant for the argument that I have been developing here. It seems to me that regardless of whether or not we also require divine forgiveness, human efforts to overturn radical evil already provide enough support for the view that we as humans we have a conditional duty of virtue to develop forgiving attitudes towards one another.

42 Note that duties of virtue are still duties for Kant, i.e. there are not any less binding than perfect duties (Wood 2009, p. 229).
